# On the Mortality of Brussels, Compared with That of Other Large Cities

**Published:** 1845-01

**Authors:** 


### ART. XIII.

*De la Mortalité à Bruxelles, comparée a celle des autres Grandes Villes.* Par ED. DUCPETIAUX, Inspecteur Général des Prisons et des Etablissements de Bienfaisance; Membre de la Commission centrale de Statistique du Royaume, du Conseil central de Salubrité publique de Bruxelles, des Sociétés Médicales de Bruxelles et de Gand, &c.—*Bruxelles,* 1844.

*On the Mortality of Brussels, compared with that of other large Cities.* By ED. DUCPETIAUX, &c. &c.—*Brussels,* 1844.

M. DUCPETIAUX is already favorably known by a long series of works devoted to the social improvement of the poorer classes of his countrymen. Since 1827 he has busied himself in preparing for the press no less than thirty publications, of which this is the last, and by no means the least important, all devoted to this excellent object. The abolition of the punishment of death, the influence of want and ignorance on crime, the improvement of the condition of the insane, the means of relieving and preventing poverty, the state of prisons, the system of solitary confinement, temperance societies, savings-banks, the physical and moral condition of young people employed in factories, the prevalence of intemperance among the working classes,—these are among the topics which have employed his unwearied pen. But while his efforts have been directed, as those of a good citizen should be, to the improvement of the physical and moral condition of his own countrymen and townsmen, he has not been unmindful of the efforts making in other countries for the promotion of the same good cause; but, with a laudable industry, has selected from the official and other records of England, France, and Germany, the most recent and most valuable contributions to the science of public health, and has brought them to bear with talent and success on the questions which have engaged his attention. The present publication contains matter which will well repay the labour of extraction. The following passage from the author's introduction sufficiently explains the aim and scope of his labours.

"The principal object of this work is to expose the deplorable condition of the labouring class at Brussels, the dangers to which it is incessantly exposed, and the necessity of taking prompt and complete measures for snatching it from the pernicious influences which decimate it. In our handsome capital there are streets, nay, entire districts, where death wields his scythe as in certain cities of the east in the time of the plague. Of two infants born there, one is inevitably condemned to perish before it has attained its fourth or fifth year. More than a third of the inhabitants die in hospitals. Without entering into an explanation of the causes which lead to these sad consequences, suffice it to state that more than a fourth part of the population is inscribed on the list of paupers, and that nearly a fourth of the families have only a single room to live in." And yet "the city of Brussels, setting aside the causes of unhealthiness which attach neither to its climate nor to its position, and which affect only the labouring class, is one of the most healthy cities in Europe. Those inhabitants who enjoy a certain independence live as long, or perhaps even longer, than in any other locality."

Such is the state of things at Brussels, and such, or nearly such, is the present history of every large city on the face of the earth. The wealthy congregated in open and airy streets and squares, in handsome and spacious houses, with every advantage and every luxury which money can purchase; the poor, crowded into narrow alleys and blind courts, or thrust into damp cellars under ground, reeking with filth and every species of abomination. The consequences of these opposite conditions are, health and long life to the one, disease and premature death to the other. Would that these were the only ones! But our author shall conduct us through some of the poorest quarters of the splendid city of Brussels. During the winter of 1838, a commission of inquiry, appointed by the central board of Public Health, and of which he was a member, minutely examined some of the worst localities, and published a Report, of which we give the following abstract.

The commissioners directed their inquiries chiefly towards the poorest and most populous districts, into which the labouring class is continually crowding more and more closely as the new and elegant structures which are springing up in the centre of the capital encroach on the localities which were originally tenanted by the poorer class alone. A great number of houses were entered and minutely examined, and their inmates interrogated as to their condition. The entire district presented an aspect of unmixed poverty. The streets and alleys, always dirty and ill-paved, become during rain and thaw, so many ponds of filth, the water having no means of escape. The passages are so narrow, there is such a complete absence of open courts or gardens, the houses are so badly built, and the population so crowded, that the circulation of air and ventilation is, so to speak, impossible. The most essential conveniences are wanting in the greater part of the houses; they have neither sewers nor privies, nor pumps, or, if there are any, they must supply the wants of large numbers of persons. In one instance, nearly seventy families were obliged to make use of the same pump and privy. On entering the houses the sight was still more painful. Though some of the rooms reminded the visitor of the proverbial cleanliness of the Flemish, they were few and far between; while the alleys, the landings, and the staircases, occupied by numerous families, were in a state of the most disgusting filthiness. The space occupied by each family was too narrow to supply the quantity of air necessary for health, their appearance, therefore, was languid and miserable, and the children, pale and attenuated, bore visible traces of premature suffering. The number affected with rickets, scrofula, and chlorosis, was considerable, and the mortality among infants and old people such as to surpass even the most unfavorable averages. When one visits these receptacles of misery, one is astonished to see so few old people, and when one questions the parents, there are few who have not to deplore the loss of one or more children.

The intellectual state of these families was not more favorable than their physical condition. But we shall not enter into the details necessary to establish this position. Suffice it to state that in every respect, physical and moral, the city of Brussels would suffer by comparison with any but the very worst districts of the worst towns of our own country; and this is saying a great deal. We beg pardon, it does not appear that any of its inhabitants live in cellars. We would not for the world deprive Liverpool of its bad pre-eminence in this respect.

We will now follow our author step by step in his careful analysis of the statistical data which exhibit the effect of these unwholesome influences on the health, life, and general well-being of the poorer inhabitants of Brussels. The population of Brussels, on the 15th of March, 1842, was 113,207, of which 52,538 were males, and 60,569 females. During the three years from 1840 to 1842 inclusive, there were 10,976 deaths, or 3658 a year, exclusive of children still-born. In round numbers, then, there was one death in 31 inhabitants, or including still-born children, one in 28½. During the eighteen years from 1825 to 1842 inclusive, the population increased from 84,004 to 116,650, and the deaths from 3146 to 4336. The number of deaths fluctuated during this period between 3022, in 1827, and 4704 in 1838. In 1832, the year of the cholera, 4676 deaths occurred; and in 1842, the last of the series, 4336. The proportional number varied, during the same period, from 1 in 28 in 1827 and 1828, to 1 in 20 in 1832, the year of the cholera. The highest relative mortality, next to this, was 1 in 21·5 in 1829. In 1842, the ratio was 1 in 25·7. These numbers and proportions are exclusive of still-born children, and comprise several deaths occurring in hospitals among persons not inhabitants of Brussels.

If we limit ourselves to the three years, from 1840 to 1842, and exclude the still-born, we have an average mortality of 1 in 31; and, distinguishing the sexes, of 1 in 29 for males, and 1 in 33 for females.

During the same period of three years, 1721 deaths occurred under five years, and 1937 above that age; being, respectively, 47 and 53 per cent. Out of 5812 deaths which took place during the three years, above five years of age, 1545 occurred in the Hospitals of St. Pierre and St. Jean. More than a fourth, therefore (1 in 3·7), of the deaths above five years of age, took place in these last resorts of destitution. If we add to these numbers the deaths taking place in other charitable institutions, and in the Depôt de Mendicité of La Cambre, which contains about 2000 paupers, we have nearly one third of the annual deaths taking place in the hospitals and hospices.

The mortality of 1 in 31 is that of the whole of Brussels; but it appears, from a series of elaborate tables prepared by our author, that in different quarters of the city, it varies within very wide limits. In the eight districts into which the city is divided, the mortality ranges from 1 in 30 to 1 in 46; and in one sub-district in which the deaths amounted to 1906, it was as high as 1 in 25. The sanitary condition of the city appears more distinctly from an exact comparison instituted between that part of the population which is placed in circumstances most unfavorable to health, and that which enjoys greater advantages. In the former, which comprises 206 streets and 66,182 inhabitants, the mortality is 1 in 29, more than half the deaths (54 per cent.) take place under five years of age, nearly a third of the deaths occur in hospitals, and there is one still-birth in 330 inhabitants. On the other hand, in the districts placed under the more favorable circumstances, which comprise 45,977 inhabitants, the annual mortality is 1 in 53, only 42 per cent. of the deaths take place under five years of age, 1 death in 8 takes place in hospital, and there is one still-birth in 460 inhabitants.

The population of Brussels is next divided into five classes: those devoted to industry and commerce, domestics and day-labourers, the members of the liberal professions, proprietors, and miscellaneous. The following are the chief results obtained from these classes; In the class of domestics and day-labourers or journeymen (journaliers), there is 1 still-birth to 125 persons; in the class designated as devoted to industry and commerce, 1 still-birth to 260 persons; while in the three groups comprising the liberal professions, proprietors, and other persons in independent circumstances, the proportions are 1 in 400, 1 in 600, and 1 in 2785. The same disparity exists between the annual mortality of the several classes under five years of age. Among domestics and journeymen, it amounts to 54 per cent., industry and commerce figures for 51 per cent., professions not specified 43 per cent., the liberal professions 33 per cent., and proprietors and men of independent means 6 per cent. Lastly, the annual mortality among the class of domestics and journeymen, exclusive of still-births, is 1 in 14; among the class devoted to industry and commerce 1 in 27; while the liberal professions, proprietors, &c. lose only 1 in 50·6. If we include the still-born, the numbers for the three classes respectively are 1 in 12·5, 1 in 24·7, and 1 in 49.

Our author next takes a glance at the statistics of Belgium, from which we deduce the following results: During the year 1841, there were, in the whole kingdom, 102,640 deaths, including 5532 still-births in a population of 4,138,382, which gives, exclusive of still-births, 1 death in 42·62 inhabitants, and 1 still-birth in 748 inhabitants. If we add the still-births, the mortality becomes 1 in 40; which is somewhat more favorable than that of the four preceding years, viz. 1 in 38. The births were to the deaths as 25 to 17½. The density of the population in the rural districts, and the number of persons receiving poor-relief, do not appear to bear any regular proportion to the number of deaths; but if we compare the seven provinces which have the largest population, relative to their extent, with the three which have the smallest, we find that, while the density of the population in the former is to that in the latter as 173 to 57, the mortality is as 1 in $37\frac{1}{2}$ and 1 in 42. The author very justly remarks, that the amount of relief given to the poor is no test of destitution, but rather of the extent of the funds appropriated to charitable purposes.

A startling fact, to which attention is very forcibly directed, is the constant increase in the proportional number of deaths which has taken place during the last twelve years in all the provinces of Belgium. Comparing the interval of 1825-9 with that of 1837-40, it appears that the civic mortality was 1 in 37 for the former period, and 1 in 32 for the latter, while the rural mortality for the two periods was 1 in 47 and 1 in 40; showing for the entire kingdom, in twelve years, an increase of nearly twelve per cent.

From some tabular comparisons instituted between the civic and rural districts of Belgium, it appears: 1. That life is generally longer in the country than in the towns. 2. That a residence in the country is particularly favorable to children of both sexes under five years of age; while, on the contrary, it seems to be injurious to males from five to twenty, and to females up to fifty years of age. 3. That whilst the female inhabitants of towns, during the interval from thirty to forty years of age, lose scarcely 1 per cent. more than males of the same age, and their mortality is less than that of males from twenty to thirty years of age, the females who reside in the country lose, from thirty to forty years of age, nearly 3 per cent. more than males of the same age. This is attributable to the extensive employment of females in the severe labours of the field.

The mortality of towns in Belgium, of which the population ranges between less than 2000 and more than 100,000, is as high as 1 in 24, and as low as 1 in 52. We have thrown these towns, amounting to 59 in number, into groups, according to the number of their inhabitants, and we find that, as a general rule, the mortality increases with the population. Thus, while the mortality in towns containing less than 5000 inhabitants is 1 in 37, that of towns containing from 50,000 to 100,000 is 1 in 33, and that of Brussels as high as 1 in 24. This rate differs from that already given, but the cause of the difference is explained in the first clause of the following summary. The author states:

1. That the city of Brussels is, of all the towns of the kingdom, that in which the number of deaths is proportionably the most considerable. During the period of four years, from 1837 to 1840, there was 1 death in 24 inhabitants. This proportion is higher than that deduced from the registers of the *état civil* for the years 1840-1-2, compared with the population, according to the census of 1842 (1 in 30·9). We must doubtless attribute that difference chiefly to the fact that, during the last period, the still-born have been deducted from the deaths, properly so called; and that the census-returns give a larger population than that which had been previously deduced from the census of 1829, added to the balance of births, deaths, and changes of residence ascertained subsequently to that period. If we add the still-born to the deaths during that same period, we shall have a yearly average of 3966 deaths in a population of 113,207 persons, or one death in 28·5 inhabitants. On the other hand, if we assume, as is probably the case, that the actual population of the majority of the other towns exceeds, as at Brussels, the figures given by the official returns, we shall find that the proportions remain nearly the same, though in general a little less unfavorable. 2. That though the relative number of deaths in the large towns, and especially in the manufacturing towns, is generally higher than in those of less importance, the situation does not exercise in that respect any appreciable influence. 3. That we must attribute the differences existing in the mortality of towns, not to general causes, such as situation, climate, elevation, the course and distribution of the rivers, &c., but to causes purely local.

Having demonstrated the high rate of mortality in Brussels, and compared it in that respect with the rural districts of Belgium and several of its towns, our author proceeds to inquire into the causes of that high mortality, and, by means of ingeniously-arranged tables, shows that there is a coincidence between the degree of indigence, the number of deaths, and all those circumstances, physical and moral, which are most to be deplored. Taking the number of indigent persons as the basis of his classification, Ducpétiaux divides the inhabitants of Brussels into four classes, of which the first consists of families having more than one half of their members inscribed on the lists of paupers, the second from a tenth to a half, the third less than a tenth, and the fourth none. It results from this comparison, that the first and most indigent class loses annually 1 in 30·3 inhabitants, the second 1 in 30, the third 1 in 39·9, and the fourth 1 in 50. The deaths in hospitals are, in round numbers, the first class 1 in 5, in the second 1 in 6, in the third 1 in 8, and in the fourth 1 in 9. The deaths at five years and under are to those above five years, for the first class as 55 to 45, for the second as 49½ to 50½, for the third 39 to 61, and for the fourth 32 to 68. But there are certain streets in Brussels in which the mortality under five years greatly surpasses that of the first class, amounting to no less than 70 and 75 per cent.

Though the indigence of the inhabitants is placed foremost among the causes of the high mortality of Brussels, intemperance, immorality, and want of cleanliness are allowed their full influence. Here, as elsewhere, want, ignorance, disease, and crime go hand in hand, each standing towards the other in the relation both of cause and effect. In illustration of the moral condition of this unhealthy population, it will suffice to state that, in the class of domestics and journeymen, 36 out of every 100 births are illegitimate; and that among entire classes of females, the illegitimate children form 73, 83, 98, and even 99 per cent. of the total number of births.

Having now displayed the sanitary condition of Brussels, we will follow the example of our author, and compare it with that of other large cities, premising that the mortality for the whole of Belgium for the year 1842 was 1 in 40·5, while that of England during the year 1841 was 1 in 46, and of France in the same year 1 in 40·9. Belgium, therefore, is in nearly the same sanitary condition as France, and both countries are much less favorably circumstanced than England. The mortality of Brussels, as we have seen, is 1 in 28·5, being somewhat more favorable than the two most unhealthy towns in England, Manchester and Liverpool; the former of which loses annually 1 in 28, the latter 1 in 28·3 of its inhabitants. A few of the continental cities present a still higher mortality. The number of deaths taking place in the hospitals of Brussels is very considerable. In London, in 1839-40, the deaths in hospitals were only 5·5 per cent of the total deaths; in Paris, in 1841, 27 per cent.; but in Brussels they amount to the enormous number of 31 per cent. The mortality under five years of age in Brussels is 47 per cent., while in London it is only 41 per cent.

These details are sufficient to show that the sanitary condition of Brussels is extremely unfavorable, as compared with that of most other large cities. It ranks by only a small fraction of a unit above our own Manchester and Liverpool.

We shall now conclude our notice of M. Ducpétiaux's praiseworthy labours by a summary of the means which he proposes for the improvement of the sanitary condition of the city of Brussels. Such a summary cannot fail to be useful at the present time, when happily the subject of public health is beginning to engage that attention to which its great importance entitles it.

The destitution, which he places foremost among the causes of the great mortality of Brussels, and which he shows to be constantly on the increase, is, as he most justly observes, not to be removed or materially diminished by alms-giving. Though the amount of the sums expended for the relief of the poor falls little short of two millions of francs a year, it produces little or no impression upon the mass of poverty with which it has to contend. And why? Because hitherto the efforts of the benevolent have been directed solely to the relief of the poor, without giving one thought to the prevention of poverty. We may double or triple the amount of our charities, but shall gain no useful result. We are merely casting our riches into a yawning gulf, which opens the wider with each fruitless attempt to fill it. Hopeless as is the attempt, we do not even combine to effect our object, but each man separately drops his mite into the abyss, vainly flattering himself that he is doing something towards closing it. Modern benevolence is a disorderly sort of personage, assuming a thousand odd shapes, and making use of a thousand odd instruments, excessively wrong-headed, marvellously independent, and having an Englishman's own horror of centralization. She inspires a host of well-meaning busybodies, who go forth one by one, armed with coal-tickets, soup-tickets, mendicity-tickets, with blankets, water-gruel, and advice gratis, for the so-called poor; and who really flatter themselves that these good things are working good, that they fill the craving stomachs and clothe the shivering frames of the really poor and destitute. Alas for our good intentions! It is almost a pity that we should not now and then be constrained to pay a visit to the gin-shop or the pawnbrokers. We should soon learn the real market value of tickets and blankets in those chosen resorts of the poverty that parades its rags abroad or cherishes them at home, for the especial edification of ladies bountiful and lay visitors. These things are not exactly written in the work of our author, but something not unlike them is to be found there. He, too, complains of want of system and unity of purpose.

In lieu of a single fund, he says, there are ten or twenty *caisses de secours;* instead of a common centre of distribution, there are almost as many centres as there are charitable associations or benevolent persons. Each man, as he says, has his own poor; that is to say, each man gives without inquiring what his neighbours are doing. What is the result? Why, that oftentimes the cunning rogue, who holds out his hand on every side, gathers a more abundant harvest than the really distressed; that the charity destined to supply imperious wants only serves to encourage an immoral traffic; that the honest artisan, sick and out of work, loathes to exhibit his own misery aud that of his family, and ashamed to beg from door to door, remains forgotten in his miserable abode, a prey to the most cruel privations, while a shameless band, always on the scent of charitable contributions, make a public exposure of their rapacity and degradation. We have seen these pretended poor sell their coal- and bread-tickets, which they had succeeded in obtaining, to gorge themselves with beer and gin at the cabaret; and if one would only take the pains to examine the registers of the Mont de Pieté, we should probably find that a great part of the clothing and blankets distributed, so to speak, by lottery, have lost themselves in the gulf dug by vice and misconduct.

Brussels, then, resembles our own large towns, not only in its excessive mortality, and in the destitution of which it is both cause and effect, but also in the effrontery and dishonest practices of those who make poverty their profession, who beg without a blush, defraud the destitute without compunction, and cheat unsuspecting benevolence, till it changes to a hard-hearted scepticism as to the very existence of the poverty which it endeavoured with so much enthusiasm to relieve. The obvious remedy for this state of things is association and centralization —a combination of individual effort, and a union of the scattered resources of charitable individuals and small societies. The use to be made of the funds so collected, and the direction which the efforts of the charitable should take, will best appear by following our author in his further enumeration of the causes of the high mortality of Brussels, and the means by which it may be diminished.

Indigence must be regarded not so much as the immediate cause of an excessive mortality as the means whereby men are brought into contact with unwholesome influences. Men do not perish simply because they are poor, but because their poverty exposes them to certain privations and certain dangers, from which persons in more easy circumstances can purchase the means of escape.

First and foremost among these dangers our author places the state of the dwellings of the working-class, which he describes as wanting in every element conducive to health. Space, air, light, water—all in defect; filth and foul odours abounding. A great number of indigent families are worse lodged, in many respects, than the animals. In favour of the latter we take hygienic precautions which are entirely neglected in the case of the former. We pull down ruinous and decayed houses only to build on their foundations elegant mansions for the rich, while the poor are crowded into the obscure, filthy, and infected houses in the immediate neighbourhood. No provision is made for the poor whom we are driving into worse habitations than those which they have left. We doubtless improve the appearance of our towns, and build some very handsome screens to shut out from our sight the misery which we have invaded, but what provision do we make for the poor whom we have displaced? When we inclose some worthless common, which is to the poor of the country what St. Giles's is to those of the metropolis—a damp unwholesome breeder of ague and fever—we are sadly scandalized, and justly so, if the rich reap all the benefits of the improvement; but when we destroy the chosen resorts of poverty in our towns, we feel ourselves under no obligation to restore aught of what we have taken away. We virtually say to the poor whom we have driven out of St. Giles's, "Your neighbours of Drury Lane and Gray's Inn are waiting with open arms to receive you. They are not at all over-crowded themselves, they have plenty of room: there is scarcely an apartment which has more than half a dozen occupants already; the centres and the corners of hundreds of Irish garrets and cellars are at your disposal for a very moderate rent. Go, and may health and prosperity attend you." But to be serious. Are we acting justly towards our poorer brethren, when we let slip such admirable opportunities of improving their condition? What should hinder the Woods and Forests from appropriating some part of the space which they clear, to wholesome houses for the poor? There is not an improvement, of the many which are constantly going on, which is not advocated on the ground of the benefit it confers on the working class: why should we not act up to our professions? Can any man point out another appropriation of space or money half so beneficial? It is not that the building of a few score of wholesome houses for the poor would make any great impression on the mass of misery-producing sickness which rages in all our large towns. An example is wanted, a model for the imitation of the proprietors of the houses of the poor who will think or can feel for their tenants. Can our authorities be better occupied, or the funds at their disposal be better spent than in setting such an example? If charitable individuals want to know how best to employ their wealth, we can tell them. There is a Society established for Improving the Dwellings of the Poor, which, if properly supported and well managed, will do more substantial and abiding good than all the pottering plans for the relief of destitution, which are daily concocted by an uninstructed and misdirected benevolence. The misery which a thousand ingenious plans cannot cure, a little plain common sense can and will prevent.

In Belgium as in England, in Brussels as in London, Manchester and Liverpool, there is one great obstacle to improvement on the large scale, an improvement only to be effected by legislation; and that is the so-called rights of property. On this subject M. Ducpétiaux shall speak for himself.

People will perhaps urge as an objection to our plans, (for the improvement of the condition of the houses of the poor,) the necessary interference with the rights of property. But is our respect for the rights of property to be carried so far as to endanger the public health and security? The rights of the proprietor are necessarily limited by the rights of society. That limit is inscribed upon nearly every page of our law. Why does it not also exist for the speculator who lets his houses to the workman and indigent? We impose rigorous conditions on the sale of commodities, we confiscate without hesitation, meat of bad quality, putrid fish, adulterated liquors, and bread below the legal weight; and we not only confiscate these things, but we punish their owners. By what strange contradiction do the proprietors of these hideous dens, of these infectious holes, to inhabit which is at least as dangerous as the use of the most unwholesome food, not only remain unpunished, but continue to enjoy a peculiar protection, and a sort of privilege, inasmuch as they are exempt from the greater part of the conditions imposed upon other proprietors. If we forbid the sale of arsenic, &c., why do we allow a host of wretched beings to perish by slow poison in the unwholesome habitations in which they are necessarily confined?

The fatal effects of the unhealthy habitations of the working class at Brussels, are aggravated by the mephitic exhalations of the open sewers and heaps of filth, which in winter, and especially in wet seasons, infect the streets and narrow and dark alleys in which the poorer classes reside. Add to this the want of proper conveniences, and of a due supply of water, and some idea may be formed of the unfavorable circumstances which affect the health of the working class. The remedies for this sad state of things are obvious. A better system of sewerage in all its parts; the filling up, or covering in, of the open arms of the river Senne, which, during summer especially, exhale an insupportable odour; an improved method of paving and cleansing; a better and more abundant supply of pure water; the establishment of warm and cold baths, and of proper conveniences for the use of the working-class; the widening of the streets inhabited by the poor, and the ventilation of churches, workshops, and schools.

Scanty clothing, and insufficient or unwholesome food, augment the evils which the poor man has to contend with, both within and without his habitation. The remedial measures which the author proposes under this head are, the abolition or reduction to a great extent of the municipal taxes on objects of the first necessity, such as meat, beer, and fuel; a strict surveillance over the sale of articles of food; the establishment of bazaars for the sale of objects of the first necessity, where the working class might purchase provisions in small quantities at a low price, without being obliged to have recourse to small shopkeepers. These bazaars or depôts might be established by the public or local authorities, or by associations of the poor themselves. Assuming that the number of persons in Brussels who could profit by such establishments, amount to 30,000, and that if each person spends merely 100 francs a year, an annual saving of nearly half a million of francs would be effected, allowing a difference of only 15 per cent. between the wholesale and retail price of provisions. By extending the same principle of combination to other articles of primary necessity, such as clothing, bedding, fuel, and light, we should realize a reform more important for the labouring classes than could be effected if the greater part of the plans daily suggested for their advantage were put in practice.

The next group of evils pointed out by M. Ducpétiaux, are excessive labour, the unhealthiness of certain employments, and the bad state and deficient ventilation of many of the workshops. These evils affect especially the health of women and children engaged in sedentary occupations. The remedies suggested are, the enactment of a law to regulate the labour of young persons, and for the superintendence of workshops; the revision and consolidation of the laws affecting unhealthy manufactures and employments, the purification of places of work, and the security of the working-class; the establishment of public gratuitous schools of swimming and gymnastics; the institution of popular games, and the formation of open spaces for exercise and amusement.

The habits of intemperance to which the poorer classes are addicted are next mentioned among the causes of their high mortality. The causes to which this sad habit are to be traced are thus detailed: the bad example to which they are, even from infancy, exposed; the choice of, or apprenticeship to, an occupation numbering a great many drunkards; the habit of debauch and disorder which results from the organization of clubs among those employed in the same manufacture; the complete idleness of the Sundays and holidays; the low price of brandy, gin, and other spirituous liquors, and the great number of public-houses where drinking goes on at all hours of the day and night; and lastly, the absence and forgetfulness of moral and religious principles. The remedies suggested will be readily deduced from this enumeration. They are, the removal of infants and young children from the contagious example of their parents, by means of a good system of popular instruction, requiring their attendance at school; the separation of apprentices, as much as possible, from the company of the older workmen, especially where they are addicted to habits of intemperance; the prevention of the complete idleness of the Sundays and holidays, by means of instructive and amusing popular occupations, which may be conducive to morality, and consequently to comfort; the suppression of the fatal habit of absenting themselves from work on the Monday; increasing the tax on spirituous liquors, and the stronger kinds of malt liquor; closing the public-houses at an early hour, and putting a stop to the gambling going on there on the Sunday and Monday; the careful and regular publication of all the sanguinary quarrels, crimes, and accidents, occasioned by drunkenness, in order to show the people, on every occasion and by every means, all that this vice has in it of hideous, and fatal, and brutal; and especially the revival of religious feeling and practice; and the punishment of drunkenness, and its severe discouragement by all the employers of labour. Such are some of the more general causes affecting the health of the labouring class, and such the remedies proposed for their mitigation or removal. The immorality which accompanies them is illustrated by a familiar test, the number of illegitimate children brought into the world by females belonging to the lower classes of the people. We are told that in the class of semstresses, laundresses, florists, dress-makers, weavers, lacemakers, and embroidresses, which comprises at Brussels 9000 women and young girls, there is scarcely one legitimate birth in 100. Desertion of children, and an excessive infantile mortality are the necessary consequences. It appears that of the children annually admitted into the Hospice des Enfants Trouvés, from three fourths to somewhat less than a half die during the first year. In 1840, there were 572 admissions, and 301 deaths under one year; in 1841, 604 admissions, and 297 deaths; and in 1842, 606 admissions, and 257 deaths.

Education and its defects, and the want of a good medical organization to carry out the proposed reforms are next commented on by our author, who concludes this part of his subject by proposing a system of combination, by which the poor might be provided on moderate terms with wholesome houses, and all things conducive to their comfort and well-being.

We would refer those who are interested in this most important subject, to the appendix of M. Ducpétiaux's work, in which will be found detailed plans with illustrative engravings. We shall content ourselves with merely indicating the chief objects which he proposes to accomplish. We know, he says, in what way the working-class is lodged, fed, and warmed; the employment of the means which we have pointed out, would, beyond a doubt, improve his position in these respects; but that improvement would be much more complete, and much more real, if we were to substitute for those filthy, damp, dark, and ill-ventilated ruins into which the poor are now crowded, houses as spacious as possible, agreeable, commodious, well-lighted, and well-ventilated. These houses might be built in the faubourgs, where land, building materials, and provisions are at a lower price. They should be arranged with regularity, in such a manner as to form streets, and entire quarters, varying in extent according to the number of persons, and the amount of their resources. In addition to their separate habitations, there should be places in common, a kitchen, a washhouse, an infirmary, baths, a common room for social meetings, an infant school, and a large open space planted with trees, with a portion set apart for games and gymnastic exercises. If the ground was moderate in price, we might attach to the quarter a certain number of small gardens.

Such are the evils which press upon the labouring population of Brussels, and such the remedies which our author would employ to mitigate or remove them. He concludes his very useful and able work by an appeal which would lose none of its force were it addressed to the wealthy and influential inhabitants of our own large towns.

"On recapitulating," he says, "the remedies to be applied to the excessive mortality of the working classes of Brussels, we have wished particularly to reply to that eternal argument of the indifferents and pessimists, who, when we point out any abuse or suffering, affirm at once that we must tolerate that which we cannot prevent, and that it would be a useless and foolish task to attempt to dry up the source of the evils which afflict humanity. Of all the means which we have proposed, there is not a single one which is not perfectly practicable, and which if applied, would not afford a real relief to the sufferings of a class whose lot is so deserving of pity. These means, nevertheless, are simple, and not expensive, especially when we have regard to the important object to be accomplished. They consist in a more useful and rational employment of the large resources which are annually absorbed in the capital, without any other result than that of maintaining indigence at its habitual level, and perpetuating the dependence and degradation of the unfortunates obliged to have recourse to public or private charity. We have seen that these resources may be valued at nearly two millions of francs; the appropriation every year of a part only of this sum to the work of prevention in the manner already pointed out, would suffice to reduce the enormous charges which the several charities impose. We should in this way reduce not only the budget of indigence which presses upon all the inhabitants, but the budget of suffering and mortality which presses almost exclusively on the labouring population. With this double advantage in view, it appears to us impossible that we can long remain indifferent. Almost every hour which strikes in our handsome capital corresponds to the last struggle of a dying man. If we take, as the average mortality of Brussels, that of the intermediate class, which, without enjoying independence, properly so-called, is nevertheless exempt from the privations which afflict exclusively the lower class, the figure of the mortality ought not to exceed the population of one death to forty-five inhabitants. By employing the means which we have passed in review, we have no doubt that we should attain that average for the entire population. We should thus save, every year, nearly 1500 wretched beings who now succumb to the influence of the deadly causes which are perpetuated under our very eyes. We adjure our municipal authorities, whose intentions are so pure and generous, we adjure all those of our fellow-citizens who, like ourselves, sympathize with the sufferings of the working class, to listen to our words. The question which we have discussed is one of life and death; we might have disguised the truth, but we have preferred openly displaying it, sure of being understood by some chosen spirits, who would not hesitate to join their efforts to ours to change a state of things, which if it should continue any longer, would impugn our civilization, and throw upon each of us a very grave responsibility."

We have given thus much space to M. Ducpétiaux's work, and have made much use of his own words, because the subject is one not of local but of general interest. It is not as the capital of Belgium, but as a large town, not as a special case, but as a general illustration, that we attach importance to the details of its mortality, of its insalubrity, and of the means of diminishing the one, and removing the other. If for Brussels you read London, Birmingham, Manchester, Liverpool, Glasgow, or Limerick, you will scarcely discover your mistake. The description of one large town may stand for that of another. The figures which represent the mortality may differ by a few units. The map may be shaded a little deeper, there may be more distress, more intemperance, more ignorance, more crime in one large town than in another, but the difference in degree will appear only in the tables, and the same words may be applied, without fear of exaggeration, to the best and to the worst. Each large city has its own peculiar mode of doing injustice to the poor: one delights in cellars and blind courts; another encumbers every available plot of ground with houses, without leaving a single open space for exercise and recreation; a third rejoices in an unusual scarcity of water, or exhibits a laudable economy in the matter of sewerage. All agree in shutting their eyes as long as they can to the evils which they are inflicting upon the poor, and in setting about the work of improvement as slowly and lazily as possible. The broad example of ignorance and bad economy set them by the government, is followed and improved upon by the municipal authorities, and thus, amid the slumbering idleness and quiet indifference of wealth and power, the poor man lives out his life of wretchedness, and wends his way unheeded and forgotten to his early grave.

The parallel between the present condition of the city of Brussels, and that of our large towns will be found to hold good in every particular. There, as here, the mortality is excessive, and the duration of life shortened by several years; and in both countries the same causes are in constant operation. The houses of the poor are badly constructed, scantily provided with water, and destitute of the means of cleanliness and decency. Ventilation is entirely neglected in all their places of resort. The workshop, the school, the hospital, and even the church are reeking with foul air. They are confined in the unwholesome shop or factory during long hours of work. The streets are without sewers, and filled with stagnant pools or heaps of filth. There are no open spaces for exercise and recreation, no baths for cleanliness. The mind of the poor man is as much neglected as his body, and vice and intemperance come as naturally to him as disease. How should it be otherwise?

Then there are the same obstacles to improvement here as in Belgium. The mass of mankind, of course, is both ignorant and indifferent, but those who really desire to help the poor begin at the wrong end. Instead of preventing poverty, they waste half the resources at their command in abortive attempts to relieve distress; and find with regret that every ingenious invention to prevent imposition is met with a still more ingenious plan to rob the distressed of the bounty which was intended for them. Instead of growing wise by failure, the philanthropist too often resigns himself to despair, and thus the poor are robbed one by one of those who had the best inclination to serve them. Experience of the little advantage derived from individual efforts leads to the formation of societies; but these have, though in a less degree, all the faults and defects which attach to the solitary efforts of the philanthropist. Great evils demand large resources, and these can only be obtained by a combination of many individuals. This spirit of combination for the carrying out of great and comprehensive plans for the benefit of the poorer classes is only beginning to work, and the want of it heretofore has been among the chief obstacles in the way of their improvement.

The right of property and the national horror of centralization are two other stumbling blocks in the poor man's path. Any man who has accumulated capital enough to hire the services of a workman, or to become the proprietor of a house may, with impunity, do what he pleases with either. He may overwork the poor wretch as much as he chooses, and compel him to breathe any kind of atmosphere; and he may let his house in any condition which seems best in his own eyes. These are the so-called rights of property, which we regard with such profound respect, that to touch them seems a sort of sacrilege. But, happily for the poor man, his wrongs are sometimes weighed against these rights, so as to strike a balance in his favour; and, happily for the rich, the curtailment of their assumed rights generally turns to their own advantage; so that, little by little, this obstacle is being removed, and threatens to leave the bugbear of centralization, in all its appalling magnitude, to fright the isles rom their propriety.

A word about centralization. There are some things which can only be well done, when done on a large scale. They not only require large means, but unity of purpose. Drainage, and sewerage, and the supply of water are of this nature; and they are precisely those things which most conduce to the health of all classes of society. If intrusted to local boards, they are sure to be badly and expensively done. What real obstacle exists to the amalgamation of all these costly commissions? A central board, with a skilful staff of officers, and comprehensive plan of operations, would do double the work at half the cost, and do it much better too. But, somehow or other, we Englishmen seem to have fallen into a habit of attaching vast importance to local governments and local boards. Our very liberties appear to depend upon their maintenance and extension. The vulgar squabbles of parish authorities are as music in our ears, and there is a smack of liberty in all their jobs. We have no intention of bringing down upon us the representatives of the rights of property, and the friends of local government; we shall therefore take refuge in one or two simple questions, which we submit with all the gravity in the world. Would not our liberties be safer in the hands of a people, conscious that their health and well-being were the constant object and aim of our legislation than in all the local boards and commissions in the world? Might not a large and comprehensive system of prevention be found to answer better, and be less costly, than isolated attempts at relief and cure? Would not schools and churches prove cheaper than prisons, pure air than physic, wholesome houses and workshops than hospitals and dispensaries; a population comprising a due proportion of adults and old men—of strength and wisdom—than one of which so large a part consists of widows and young children?

To conclude: Disease is a very costly thing, both to individuals and the public; and funerals are just as bad economy as any other item of unnecessary expenditure. Health and strength are a nation's best possessions in peace, and her surest defence in war. In both, the power of making great, rapid, and continuous efforts is at least as important as the possession of ingenious machinery and powerful artillery; and the time, perhaps, is not far distant when the cost of provisions and mechanical skill and dexterity shall be so nearly equalized, that superiority shall mainly turn on the strength and power of endurance of the mechanic and the soldier; and that nation which has best husbanded its living resources shall be most prosperous in peace, and most certainly successful in war.

